# Shaping research for people living with co‐existing mental and physical health conditions: A research priority setting initiative from the United Kingdom

**DOI:** 10.1111/hex.14044

**Published:** 2024-04-13

**Authors:** Olivia Taylor, Elizabeth Newbronner, Helen Cooke, Lauren Walker, Ruth Wadman

**Affiliations:** ^1^ Department of Health Sciences University of York York UK; ^2^ Public Contributor Leeds UK; ^3^ School of Health & Psychological Sciences City University of London London UK

**Keywords:** mental illness, multimorbidity, physical health, public involvement, research priorities

## Abstract

**Introduction:**

Those with severe and enduring mental ill health are at greater risk of long‐term physical health conditions and have a reduced life expectancy as a result. Multiple factors compound this health inequality, and the need for setting research priorities in this area is highlighted with physical and mental healthcare services being separate, and limited multimorbidity research.

**Methods:**

The aim of this exercise was to work in partnership with healthcare professionals and carers, family, friends and individuals with lived experience of both mental and physical health conditions, to set research priorities to help people with mental health conditions to look after their physical health. The exercise was guided by the James Lind Alliance approach. For this, a steering group was set up, two surveys were completed and a final priority workshop was conducted.

**Results:**

This priority setting exercise guided by people's needs and lived experience has produced a set of well‐defined research topics. Initially, 555 research questions were suggested in the first survey, which were refined to 54 questions for the second survey. A priority setting workshop was then conducted to get the final 10 priorities.

**Conclusions:**

Taking these topics forward to improve services and treatment for both mental and physical ill health may in turn improve physical health and lessen the reduced life expectancy of those living with mental ill health.

**Patient or Public Contribution:**

This work was completed in collaboration with people who have lived experience of mental ill health and physical health conditions, as well as carers, family and friends. Their contribution has been significant for this work from piloting surveys, amending language used and educating the researchers and contributing to this paper. The initial work was completed with a steering group and continued with surveys and workshops.

## 1 INTRODUCTION

Successive studies have shown that people with severe and enduring mental ill health are at greater risk of developing long‐term physical health conditions such as cardiovascular disease, respiratory and metabolic conditions and cancer.^1^, ^2^, ^3^ Consequently, they are likely to live with poor physical health for a greater proportion of their life and to have a substantially reduced life expectancy compared to the general population.^4^, ^5^, ^6^, ^7^ This gross health inequality has been described as both a public health scandal^8^, ^9^ and a human rights issue.^10^

The factors associated with higher morbidity and mortality rates in people with severe mental ill health are already known. Whilst commentators describe them in different ways and place emphasis on different factors, the common elements are clear. Living with mental ill health can have a significant adverse impact on people's ability to maintain a healthy lifestyle.^11^ In particular, an increased likelihood of health risk behaviours such as smoking, poor diet and physical inactivity^12^, ^13^, ^14^, ^15^; difficulties in taking up and accessing preventative services such as screening services^16^ and routine dental care^17^; difficulties associated with the practical, emotional and physical complexity of managing multiple health conditions.^18^, ^19^ In addition, some antipsychotic and antidepressant medications have metabolic side effects such as weight gain.^20^ Furthermore, people with severe mental ill health are more likely to be disadvantaged socioeconomically^21^ and such inequality is known to accelerate multimorbidity.^22^

The separation of mental and physical health care, common in most higher income countries such as the United Kingdom, hinders the integration and coordination of care for people with multiple mental and physical health conditions.^23^ In particular, there are concerns that mental health and primary care practitioners pay insufficient attention to the physical health of people with severe mental ill health.^9^, ^24^ Additionally, it has been suggested that greater specialisation of healthcare professionals, especially hospital‐based doctors, disadvantages people with multiple and seemingly unrelated diseases.^25^ It may lead to multiple unrelated interactions with the healthcare system, limited understanding of the impact of severe mental ill health on people's ability to manage their physical health, stigma or diagnostic overshadowing and ultimately poorer outcomes.^26^, ^27^

In the United Kingdom, health policy has long recognised the importance of improving the health of people living with both mental and physical health conditions.^28^, ^29^, ^30^ However, some have argued that research has not kept pace with and supported this aim. One study found that there was limited multimorbidity research capacity in the United Kingdom.^31^ Whilst others note that ‘Despite the increasing amounts of research in this area and more general advancements in healthcare and medicine, the poor physical health outcomes (and associated decrease in life expectancy) of people with mental illness have not improved’ (p. 676).^10^ Similarly, Mercer et al. suggested that ‘research into multimorbidity requires a shift in design, funding and outcomes of interest’ (p. 1). Part of this shift should be greater involvement of people with lived experience of co‐existing mental and physical health conditions in shaping research priorities.^32^

The aim of our study was to work in partnership with people with lived experience of co‐existing mental and physical health conditions, family carers and health and social care practitioners from across Yorkshire and Humber, to conduct a research priority setting initiative. Specifically, we wanted to identify topics or questions that would help people with mental health conditions look after their physical health, and what treatments and services, if examined by research, could make a real difference to people's lives. This paper describes the findings from the research priority setting exercise, using the REPRISE (Reporting Guideline for Priority Setting of Health Research) guidelines for reporting priority setting in health research.^33^ It suggests priorities for future research into mental and physical multimorbidity and highlights lessons learnt from the process used.

## 2 MATERIALS AND METHODS

### 2.1 Context and scope

Yorkshire and the Humber region covers almost 15,500 km^2^ and is home to 5.2 million people. Over 80% of the population live in urban communities, but there are also large rural areas. The region ranks the third lowest in England for life expectancy, in both males and females, and around one in five residents live in geographical areas that are classified as being in the most deprived decile of England.

Yorkshire and Humber Applied Research Collaboration (YH ARC) was set up to support research designed to tackle health inequalities and improve health across its communities. It is funded by the National Institute for Health and Care Research (NIHR) for England. The Mental and Physical Multimorbidity Theme, one of YH ARC four main themes, focuses on addressing the challenges of living with and treating mental ill health and physical health conditions. The aim of the priority setting initiative was to identify priorities for future research from the perspective of people living with both mental and physical health conditions (including family carers), and the health and care professionals who support them. The initiative focused on adults (i.e., 18 and over) with a primary mental health condition and physical health conditions, and encompassed:

1. Promoting the health and wellbeing of people with mental and physical multimorbidity.
2. Treating and managing (including self‐managing) co‐existing mental and physical health conditions.
3. Improving services and support for people with both mental and physical health conditions, and their families.

Although the focus was on Yorkshire and Humber, it was hoped that the results would be of interest to researchers, research funders and mental health service providers from across the United Kingdom.

### 2.2 Governance and team

Plans for the project were developed by the YH ARC Mental and Physical Multimorbidity team, who then brought together a multidisciplinary steering group to oversee and co‐produce it. The group began by agreeing on the protocol for the project, including its scope and proposed framework for the priority setting process. The steering group included public contributors, mental health practitioners and researchers from the YH ARC Mental and Physical Multimorbidity team. None of those involved had prior experience of priority setting. However, the researchers were able to seek advice from colleagues in another YH ARC team whose priority setting project was further advanced. A total of four meetings were held with the steering group between August 2020 and September 2021. Due to the coronavirus disease 2019 (COVID‐19) pandemic restrictions, all the meetings took place via Zoom.

### 2.3 Framework for priority setting

Internationally, there are a number of recognised frameworks for conducting research priority setting. In the United Kingdom, the James Lind Alliance (JLA) priority setting partnership (PSP) approach is widely used and highly regarded. It was developed within medical research to help patients, carers, clinicians and practitioners to work together and agree research priorities for particular medical conditions (http://www.jla.nihr.ac.uk/about-the-james-lind-alliance/). As our priority setting initiative was broader in scope, we used an adapted version of the JLA approach. This comprised five stages (rather than the seven set out by JLA):

1. *Stage 1*: Initiation and consultation.
  Set up steering group; agree scope/approach and initial questions; pilot questions; report back to steering group and agree final questions.
2. *Stage 2*: Identifying unanswered questions (data collection).
  First online/paper survey; review research priorities identified from existing reviews and initiatives.
3. *Stage 3*: Initial analysis and evidence checking.
  Data cleaning; thematic analysis; present draft long list of priorities to steering group; mapping existing evidence against priorities.
4. *Stage 4*: Interim priority setting.
  Create online survey; pilot and refine survey questions; run survey; analyse survey results.
5. *Stage 5*: Agreeing on research priorities and planning next steps.
  Run final priority setting online workshop; Prepare report from project.

The main differences from the JLA approach were that (due to resource constraints) we did not have a separate stage for evidence checking, and we modified the final stage to combine the final workshop and preparation of the report from the project.

### 2.4 Stakeholders or participants

The steering group comprised: four public contributors (three people with lived experience and one family carer); three mental health practitioners; and three researchers from the YH ARC Mental and Physical Multimorbidity team (E. N., O. T. and R. W.). Public contributors were remunerated for their involvement, and we provided support and training for online meetings and activities. As part of the process of collecting information about potential research priorities, we sought input from people with lived experience, health and care practitioners and voluntary and community sector staff. Although the focus was on Yorkshire and Humber, we did not exclude participants from other parts of the United Kingdom.

### 2.5 Identification and collection of research priorities

The identification and collection of potential research priorities involved two main activities—a short survey and a focused examination of existing reviews, key papers and other documents about mental and physical multimorbidity. These are described in more detail below.

#### 2.5.1 First survey: Development and data collection

The first step in gathering ideas about possible research priorities was to develop a survey. Working with our steering group, we looked at examples of surveys used by other JLA PSPs and then drafted the questions for our survey. After some refinement of the wording, we agreed three questions:

1. What questions about how to look after your mental and physical health (or that of your relative/patient) would you like to see answered/explored by research?
2. What questions about treatment and services for people with both mental and physical health problems and/or their families, would you like to see answered/explored by research?
3. Thinking overall about supporting people with both mental and physical health problems and their families, what do you think are the most important topics for research to look at?

Each question was followed by a free text box so that the survey respondents could write as much or as little as they wished. The survey was anonymous, but respondents were asked to provide some brief biographical information, including gender, age group, ethnic background, if they lived in the Yorkshire and Humber region and whether they were: a person living with a mental and physical health condition; a family/friend carer; a third‐sector worker; or a health and social care professional. The survey was piloted (online) with four people with lived experience, all of whom were identified via the steering group members. On the basis of their comments, further minor changes, mainly to the wording of the introductory page, were made. A copy of the final survey can be viewed in the Supporting Information.

The data collection took place between October and December 2020, when the United Kingdom was still subject to COVID‐19 pandemic restrictions. For this reason, the survey was primarily online, although people could contact the research team and request a paper copy (including a large print version) and a freepost reply envelope. The survey was hosted on a secure online platform used by the University of York. Information about the priority setting initiative and the survey was distributed via NHS Mental Health Trusts in Yorkshire and the Humber; third‐sector organisations working with people with mental ill health; service user and clinical networks, and publicised on social media and in newsletters (e.g., National Survivors User Network).

#### 2.5.2 Analysis of the survey responses

The free text responses to the main questions were downloaded to an Excel spreadsheet for checking and cleaning. One researcher (E. N.) removed any responses that were unintelligible or clearly out of scope of the project. Responses that were borderline were kept in but highlighted for discussion with the steering group. Simple thematic analysis^34^ was used to identify an initial set of nine themes, representing potential research priority areas. The raw responses were then grouped under these themes. The same researcher (E. N.) reviewed the responses under each theme and removed responses that suggested the same question or topic. A second researcher (L. W.) then reviewed the responses in each theme and suggested ways in which very similar questions/topics could be combined. The questions/topics remaining were then shared with the steering group. The JLA guidance suggests that ideally a maximum of around 60 indicative questions should be included in the interim prioritisation process. Building on feedback from the steering group, the researchers shaped a final long list of questions/topics.

#### 2.5.3 Priorities identified from existing reviews and reports

The JLA recommends that as a minimum, recent reviews and resources (e.g., NICE Guidance) should be checked to see whether any emerging questions/topic have already been addressed and to identify any potential research priorities that did not emerge from the data collection. We identified seven existing reviews^10^, ^11^, ^18^, ^23^, ^31^, ^32^, ^35^ and key papers/documents about mental and physical multimorbidity that were particularly relevant to this research priority setting exercise. We examined the long list of questions/topics against these, and whilst we removed a few questions/topics (as having been addressed), we also added three questions.

### 2.6 Prioritisation of research topics/questions and outputs

There were two stages to the prioritisation process. A second survey was undertaken, in which respondents were asked to select their top 20 research questions. This was followed by a final priority setting workshop. Workshop participants discussed the priorities emerging from the survey, both from healthcare professionals and people living with co‐existing mental and physical health conditions, and agreed on the top 10 priorities. Both stages are described further below.

#### 2.6.1 Second survey: Development, data collection and analysis

At the end of the previous stage, a final long list of 54 questions/topics was agreed with the steering group (see Supporting Information), for inclusion in the prioritisation survey. Again, we looked at the resources on the JLA website to help us think about the best method for prioritisation (e.g., choosing the 10 most important questions by ranking or scoring questions). Following discussion with the steering group, it was agreed that the second survey should ask respondents to simply choose their top 20 questions (but not rank them) from the long list. In addition, respondents were asked to provide the same anonymous biographical information as was collected in the first survey.

The second survey was entirely online. Once again it was distributed via NHS Mental Health Trusts in Yorkshire and the Humber, third‐sector organisations working with people with mental ill health, service user and clinical networks and publicised in newsletters and on social media. It was open for 8 weeks during July and August 2021 for participants to respond anonymously.

#### 2.6.2 Results of the prioritisation survey

The responses to the survey were downloaded into an Excel spreadsheet. The top 20 questions were identified by simply counting the ‘votes’ for each question. No weighting was applied. The top 20 questions emerging from the survey were taken forward to a final priority setting workshop. The first 10 questions selected from the survey indicate that order bias was not a problem, as they were selected from across all 54 priorities.

#### 2.6.3 Priority setting workshop

The research priority setting workshop took place via Zoom in November 2021 and lasted 3 h. There were 23 attendees who described themselves as: a person who has lived experience or is a public contributor (*n* = 10); a family carer (*n* = 3); or a healthcare professional (*n* = 10). Five were members of the steering group. Before the workshop, attendees were sent a brief overview of the research priority setting initiative and details of the top 20 questions/topics that emerged from the second survey. Participants were recruited via our steering group, members of our theme LEAP, public contributors from other projects who had expressed an interest in the initiative and interested clinicians from partner Trusts in our region.

Following a short introduction and presentation about the research priority setting process, participants were split into four smaller break‐out ‘rooms’ with a facilitator attached to each group. Participants were asked to discuss the top 20 questions/topics identified in the prioritisation survey and then select their top 10. In the plenary session that followed, each group presented the outcomes of their discussion, and the top 10 priorities were recorded on Jamboard (a digital interactive whiteboard). Whilst there was much common ground, there was no clear consensus about the top 10 priorities. Attendees highlighted where they felt questions/topics could be merged or grouped in some way. Based on their comments and suggestions, they asked the researchers to draft their top 10 research priorities and circulate them to workshop attendees for comment. The final top 10 research priorities are described in the results section below.

### 2.7 Evaluation and feedback

Informal feedback was gathered from the steering group about their experience of being involved in the research priority setting initiative. In addition, all the priority setting workshop attendees were invited to complete a short feedback form. The top 10 priorities have been shared with NHS partners and other stakeholders from across Yorkshire and Humber in meetings, via email and on the YH ARC website.

## 3 RESULTS

### 3.1 Initial survey

There were 103 responses to the initial survey (including just one by post), with the majority (71.8%) of these coming from people living or working in the Yorkshire and Humber region. There was a good balance between people with lived experience and their family/friends, and health, social care and third‐sector professionals and a spread across the age groups. However, three‐quarters of the respondents were female and people from non‐White ethnic backgrounds were underrepresented. Table 1 below provides a more detailed picture of the respondents for both the initial survey and the prioritisation survey.

**Table 1:** Survey 1 and 2 respondents demographics.

	Survey 1 (*n* = 103)	Survey 2 (*n* = 107)
Background		
Charity/third‐sector worker	1	1
Health or social care worker	35	40
Family, friends or carer of someone living with physical and mental health problems	15	13
Person living with mental and physical health conditions	38	40
Other	14	12
Age		
16–24	1	2
25–39	27	22
40–54	34	43
55–70	32	31
70+	9	8
Did not answer		1
Gender		
Male	26	27
Female	74	77
Prefer not to say	2	1
Prefer to self‐describe	1	2
Area		
Yorkshire and Humber	74	99
Other	29	8
No response		
Ethnicity		
Asian/Asian British	5	4
Black/Black British	1	3
Mixed ethnic background	4	3
Other	4	1
White	86	92
No response/prefer not say	3	4

The 103 respondents submitted 555 questions/topics. After removing duplicates, out‐of‐scope questions/topics and responses that were unintelligible, this fell to 312. Following the review by a second researcher and the merger of similar questions/topics, 190 questions/topics remained. The steering group then highlighted where they felt there was further scope for combining or even removing questions/topics. They also suggested that some of the themes could be merged/reframed. At the end of this process, 54 questions were taken forward into the prioritisation survey. The full list of questions can be seen in Supplementary Materials.

### 3.2 Prioritisation survey

One hundred and seven people responded to the prioritisation survey. The profile of respondents was similar to the initial survey, with slightly over half the respondents describing themselves as a person living with or a family/friend of someone living with physical and mental health issues. Again, men and people from non‐White ethnic backgrounds were underrepresented (see Table 1 above). There was a remarkable degree of consensus between people with lived experience and family/friends, and health and social care professionals and third‐sector workers about which questions should be in the top 20, with 13 questions being selected by both groups. Table 2 below shows the top 20 questions for both groups and overall. The order of the questions is based on how many people included the questions in their top 20.

**Table 2:** Top 20 priorities from Survey 2.

Final order	Interim survey results		Question
	People with lived experience/family carers	Health/social/VCS practitioners	
1	1	1	How can mental and physical health services best work together to coordinate care and support for people with both mental and physical health issues?
2	3	2	Some people are living with mental health issues and long‐term physical health conditions. What is the best way to support and treat their conditions together rather than addressing each one separately?
3	4	8	How can the challenges of navigating several different health services e.g. dealing with multiple appointments and information requests, be reduced or made easier?
4	2	4	Can effective pain management improve peoples' mental health?
5	7	7	What are the major barriers for people with mental health issues when accessing physical health services and how can these be overcome?
6	9	6	How can people be supported to look after their mental and physical health when they face high levels of deprivation and poor access to services?
7	19	3	How can the social isolation experienced by people living with mental and physical health issues be reduced or better managed?
8	10	11	How can patients and their friends or family carers be supported in their understanding of how mental health issues can impact physical health issues and how physical health issues can impact mental health issues?
9	5	13	How can weight gain linked to medication(s) be reduced or avoided?
10	20	5	How can a better understanding of mental health issues be created in physical health services and a better understanding of physical health problems be created in mental health services?
11	14	12	How can conversations between GPs and people living with mental health issues be improved when discussing their physical health?
12	13	20	What is the best way to support people in maintaining their physical health when they are facing the challenges of mental health issues, low motivation or struggling to concentrate or remember information?
13	20	10	What are the most practical and sustainable ways to treat sleep problems experienced by people with both mental and physical health issues?
14	6	33	How can self‐management support for long‐term physical health conditions be made more accessible to people living with mental health issues?
15	24	9	How can services reach the most vulnerable groups of people with mental and physical health issues (e.g., those who are homeless, those in disadvantaged communities)?
16	16	35	How can overprescribing medications and prescribing medications that react negatively with one another be reduced or eliminated?
17	8	17	Would new specialist services for people living with severe mental health issues and long‐term physical health conditions make a difference to their overall health?
18	15	14	Can providing healthy meals (e.g., meals on wheels) and/or supporting people to cook healthy meals (e.g., cooking coaching; access to low‐cost cooking equipment) help people with severe mental illness manage their weight and related physical health conditions (e.g., diabetes)?
19	12	42	Could regular support from a physiotherapist or personal trainer be of benefit to people living with mental and physical health issues in helping them to become more active?
20	19	27	What are the barriers to people with mental health issues using schemes to help with physical health issues (e.g., leisure cards, social prescribing, gym prescriptions)?

Abbreviation: VCS, voluntary and community sector.

The survey results show complete agreement for the first research priority across both groups. The top five research priorities also came within the top 10 for both groups, indicating a general consensus for the research questions of the highest importance. However, there were also a number of interesting differences.

Health and care professionals collectively placed the question—‘How can the social isolation experienced by people living with mental and physical health issues be reduced or better managed?’ at number 3, whereas people with lived experience/family carer, it came in at 19. Similarly, they placed the question ‘How can services reach the most vulnerable groups of people with mental and physical health issues (e.g. those who are homeless, those in disadvantaged communities)?’ at number 9 compared to 24 for people with lived experience/family carers.

Conversely, people with lived experience/family carers placed the question ‘How can self‐management support for long‐term physical health conditions be made more accessible to people living with mental health issues? ’ at number 6, whereas health and care professionals placed it at 33. Similarly, the question ‘How can over‐prescribing medications and prescribing medications that react negatively with one another be reduced or eliminated?’ came in at 16 compared to 35 for health and care professionals, and the question ‘Could regular support from a physiotherapist or personal trainer be of benefit to people living with mental and physical health issues in helping them to become more active?’ at 12 compared to 43, respectively. We consider possible reasons for these differences in the Discussion below.

The steering group and workshop attendees decided that the final top 10 questions to emerge from the priority setting workshop should not be placed in a ranked order but instead are grouped under four meaningful headings or themes. A draft of the top 10 questions, formatted in this way, was circulated to all the workshop attendees and their comments were incorporated into the final version. The final list of questions under each heading is shown below in Box 1:

Box 1 **Top 10 research questions**

*Coordination of care and access to services*

1. Some people are living with mental health issues and long‐term physical health conditions. How can:

- Mental and physical health services best work together to coordinate their care and support?
- The challenges of navigating several different health services, for example, dealing with multiple appointments and information requests, be reduced or made easier?
- Their conditions be cared for and treated together rather than each one being addressed separately?

2. How can people with mental and physical health issues, including people in vulnerable groups (e.g., those who are homeless, those in disadvantaged communities), be supported to look after their mental and physical health when they face high levels of deprivation and poor access to services?

3. Would specialist services for people living with severe mental health ill health and long‐term physical health conditions make a difference to their overall health? What can we learn from current ‘best practice’ about how to organise and deliver specialist services?

*Understanding the link between mental and physical health*

4. How can a better understanding of mental health issues be created in physical health services and a better understanding of physical health problems be created in mental health services?

5. How can conversations between GP's and people living with mental health issues (including annual health checks) be improved when discussing their physical heath?

6. How can patients and their friends or family carers be supported in their understanding of how mental health issues can impact physical health issues and how physical health issues can impact mental health issues?

7. Can effective pain management improve peoples' mental health?

*Managing medication*

8. The side effects of medications and the interaction between medications are a major concern for people living with mental health issues and long‐term physical health conditions. How can:

- Side effects linked to mental health medication (e.g. weight gain, dry mouth/dental problems) be reduced or avoided?
- Overprescribing of medications and prescribing medications that react negatively with one another be reduced or eliminated?

*Health promotion*

9. Can providing healthy meals (e.g., meals on wheels) and/or supporting people to cook healthy meals (e.g., cooking coaching; access to low‐cost cooking equipment) help people with severe mental illness manage their weight and related physical health conditions (e.g., diabetes) in the long term?

10. People living with mental and physical health issues often find it hard to keep physically active. How can we:

- Better understand the barriers to people using schemes to help with physical health issues (e.g., leisure cards, social prescribing, gym prescriptions)?
- Identify the most effective and ongoing ways to support people to be more active (e.g., support from a health and wellbeing coach, peer support and group sessions, making use of green spaces)?

Immediately after the workshop, all attendees were invited to provide feedback (via an online feedback form) about their experience of being involved in the workshop and the steering group (where applicable). Nine attendees completed the feedback form which focused on whether the workshop was a good use of their time and if the workshop had clear objectives that were met, all of the attendees who responded to these selected either strongly agree or agree. Attendees were also asked if the topics covered were relevant to them, of the respondents seven strongly agreed, one agreed and one selected not sure. Attendees also had the opportunity to comment on what they enjoyed most about the workshop and it was reported that the workshop was an opportunity to discuss proposed research questions; all participants were listened to equally with no academic or other biases and that it was enjoyed because of the importance of grounding research in the reality of the lives of the people whose health they aim to impact. The survey also included open questions asking attendees what could have been improved, if anything from the workshop had an impact on them and if they wanted to leave any other comments.

## 4 DISCUSSION

This research priority setting exercise brought together people living with mental and physical health conditions, family carers and health and care professionals to share their expertise and determine priority areas of research. The final top 10 priorities reflect the continued challenges that those living with co‐existing mental and physical health conditions face when navigating combined care and treatment for their health conditions. They also reveal the importance people place on practitioners and services having a more applied understanding of the link between mental and physical health, and the place of a wider societal approach to health promotion for people with mental ill health. Crucially, they suggest the need for a stronger focus on research designed to develop or implement practical solutions that can make a real difference to peoples' lives.

The first survey included a prioritisation question ‘Thinking overall about supporting people with both mental and physical health problems and their families, what do you think are the most important topics for research to look at?’ Although the JLA guidance and varied examples from the JLA website were used, some adaptations were made to fit the process within shorter timeframe and less resources due to budget constraints. Additionally, the steering group had a great deal of input into the survey, so this question reflects the strong collaboration within this work.

From the second survey, there was complete agreement on the first research priority across health and care professionals and people with lived experience/family carers. In addition, the overall top five research priorities came within the top 10 for both groups, indicating a high level of consensus around the research questions of the highest importance. However, in the overall top 20 questions, a number of interesting differences emerged. We speculate that health and care professionals may have ranked questions related to vulnerable groups or wider social issues more highly because they reflect the challenges they face in their day‐to‐day work, that is, they understand the importance of addressing wider aspects of peoples' lives, such as social isolation and poor housing, but often lack the reach or resources to do this. As such, they are perhaps taking a collective or societal view rather than an individual one. In contrast, people with lived experience/family carers tended to rank questions about specific areas of care and support (e.g., medication, weight management, and access to physiotherapy) more highly. Due to modest participant numbers, we tentatively suggest this may be partly because people with lived experience/family carers understandably give greater importance to topics or issues they have personally experienced and/or which relate to individual health concerns. However, it may also reflect people's desire for better support for self‐managing their conditions.

The survey also highlighted the importance placed on some specific research topics. Two questions in particular stand out: ‘Can effective pain management improve peoples’ mental health?’, which was placed at number 4 overall and ‘How can weight gain linked to medication(s) be reduce or avoided?’, which was placed at number 9. However, both questions were given a higher place by people with lived experience, perhaps again reflecting a desire for greater self‐management. The issues raised by these questions are fundamental to overall health and quality of life and yet appear to have attracted limited attention from the research community.^36^

In the final workshop, participants worked together to consider the similarities and differences in the top 20 questions that emerged from the second survey. The initial plan, in line with the JLA approach, had been to try and agree on a final list of the top 10 questions. However, the participants decided that a more flexible approach was needed, if the final list of questions was to encompass all the topics of importance. They also suggested that grouping questions would encourage researchers to consider related questions, for example, in relation to managing medication, where the side effects of mental health medication and the interaction of medications for different conditions are both of importance.^11^ This approach of grouping priorities was suggested by members with lived experience and worked best for this area because of the complexity of managing both physical health and mental ill health, each member shared their own experiences and highlighted the importance of each priority. The priorities were merged within the workshop utilising a Jamboard and then finalised by three researchers. They were then sent to all members of the workshop to share their comments.

To our knowledge, this is the first research priority setting exercise guided by the JLA focused on mental and physical multimorbidity in working‐age adults. Whilst the results predominantly represent those living and working in Yorkshire and the Humber, it is a large and diverse region and so there is likely to be generalisability across the United Kingdom and to other high‐income countries with similar healthcare systems. The demographic information gathered showed that people from minority ethnic groups were underrepresented in both the initial data collection and prioritisation survey and this is a limitation. However, we suggest that the topics highlighted in the final top 10 questions are relevant to all those living with co‐existing mental and physical health conditions, regardless of ethnicity. What they do not capture are the specific and additional challenges (e.g., discrimination, cultural and language barriers, etc.) faced by people from minority communities.

In terms of the research priority setting process, our adapted version of the JLA PSP approach provided a good framework. The benefits of the JLA approach were further enhanced by access to advice from colleagues who had experience using the approach,^37^ and the excellent guidance and example documents available on the JLA website. However, the process was quite time‐consuming for both researchers and the steering group members, which, combined with the lack of funding for an information specialist and external facilitation, did constrain what could be done at certain stages.

Some steering group members struggled with the volume of materials that we asked them to look at for the first survey. We were able to respond to this by arranging for some members to work in pairs and focus on a subset of responses. Steering group members also had differing levels of knowledge and confidence in using technology. When the decision was taken to hold steering group meetings online because of pandemic restrictions, we offered members training and technical support in using Zoom, and this worked well. On reflection, it would have been helpful to talk to each steering group member individually about their skills, expertise, confidence and time, and tailor their contribution at each stage accordingly.

The steering group made a substantial contribution to all stages of the priority setting process. In particular, their lived and learned experience directly informed the development of the long list of questions, significantly improving their wording and accessibility. There were challenging but useful discussions about the wording used in documents and questionnaires, particularly around the term ‘problems’ in relation to mental health with the steering group feeding back on the sensitivity of this word for people with lived experience and the importance of using ‘conditions’ or ‘issues’ instead. Importantly however, the input from the steering group and the outcome of group meetings substantially shaped the final research priorities and was rewarding both for the steering group members and for the researchers who found their perspective thought‐provoking and insightful.

Perhaps, the key measure of success of a research priority setting exercise is the extent to which it actually informs decisions about future research. Grill notes that whilst researchers frequently state the importance of involving stakeholders in projects, they remain sceptical about the benefits of involving them in research priority setting.^38^ This research priority setting initiative illustrates the value of involving people with lived experience throughout the process as their contribution ensured the final priorities were grounded by the real issues they are facing. We hope this paper will help researchers focus their research on topics that really matter to people living with mental and physical multimorbidity and the people who support them.

This exercise has been extremely beneficial for shaping current and ongoing work for the YH ARC mental health and physical multimorbidity research theme. One of the outcomes of this exercise has been plans to create an open online course aimed at healthcare professionals across the United Kingdom that focuses on managing both severe mental ill health and physical comorbidities, for example, one topic is around raising awareness for managing medications and potential interactions. Another ongoing project to take these priorities forward is a scoping review and qualitative interviews on people's experiences of service responses when having both severe mental ill health and physical comorbidities. The aim of this project is to provide services with suggestions of small cost‐effective changes that have a major impact on individuals' health and wellbeing.

## 5 LIMITATIONS

Due to COVID‐19 pandemic restrictions, we could not undertake any of the priority setting exercises face‐to‐face and surveys were online only, and we know from work on digital exclusion for people with severe mental ill health that this will have resulted in underrepresentation of those with poorer digital skills and confidence.^39^ Surveys 1 and 2 had more female‐to‐male responses; however, this was not the case within workshops. Importantly, people from minority ethnic groups were underrepresented in both the initial data collection and prioritisation survey, and participants were also predominantly from the Yorkshire and Humber area. Together, these limitations may have implications for the relevance of findings to other regions and countries. The steering group and workshop lacked external facilitation via a JLA adviser because of limited resources; however, the research team have experience in facilitating groups from patient and public involment and engagment activities and steering groups, thus understanding the importance and value that people with lived experience bring. They have a perspective we do not understand and that is respected. Careful consideration was given when planning steering groups to ensure those with lived experience were heard and all members were given the opportunity to share their thoughts. This project was guided by the JLA PSP guidance; however, adaptations were made, with one of the main ones being that at the final workshop, a list of the top 10 priorities was not agreed, instead they were merged by overlapping themes.

## 6 CONCLUSIONS

This priority setting exercise has produced a set of well‐defined research topics. The priorities that have been highlighted by healthcare professionals and those living with co‐existing mental and physical health conditions provide an outline to guide research in this area that is better aligned with their needs. Taking these priorities forward with the aim of providing services and treatment that combines mental and physical ill health may in turn improve physical health and lessen the reduced life expectancy of those living with mental ill health, who have or are at risk of developing a long‐term physical health condition.

## AUTHOR CONTRIBUTIONS

**Olivia Taylor**: Writing—original draft; writing—review and editing; project administration; visualisation; methodology; resources. **Elizabeth Newbronner**: Investigation; funding acquisition; writing ‐ original draft; writing—review & editing; supervision; methodology; validation; project administration; conceptualisation. **Helen Cooke**: Writing—original draft; writing—review and editing; methodology; validation. **Lauren Walker**: Methodology; writing—review & editing. **Ruth Wadman**: Writing— original draft; writing—review & editing; supervision; validation; methodology; investigation. All authors were involved in the priority setting activities and contributed to the preparation of the manuscript. All authors read and approved the final manuscript.

## CONFLICT OF INTEREST STATEMENT

The authors declare no conflict of interest.

## Supporting information

Supporting information.

## Data Availability

The data that supports the findings of this study are available in the Supporting Information of this article. Data sharing is not applicable to this article as no data sets were generated or analysed during the current study. Further information from the priority setting process is available as Supporting Information.
