# Supplementary material for: Shaping research for people living with co‐existing mental and physical health conditions: A research priority setting initiative from the United Kingdom
Source: Health Expect. 2024 Apr 13;27(2):e14044. doi: 10.1111/hex.14044 (PMC11015889; doi:10.1111/hex.14044)
Supplement: Supplementary file 1 — Supporting information. [file HEX-27-e14044-s001.docx]

**Supplementary Materials**

**Long List of 54 Questions**

| 1 | Some people are living with mental health issues and long-term physical health conditions. What is the best way to support and treat their conditions together rather than addressing each one separately? |
| --- | --- |
| 2 | How can mental and physical health services best work together to coordinate care and support for people with both mental and physical health issues? |
| 3 | How can a person’s mental and physical health information be shared appropriately with relevant health professionals? |
| 4 | How can a better understanding of mental health issues be created in physical health services and a better understanding of physical health problems be created in mental health services? |
| 5 | How can patients and their friends or family carers be supported in their understanding of how mental health issues can impact physical health issues and how physical health? |
| 6 | How can conversations between GPs and people living with mental health issues be improved when discussing their physical health? |
| 7 | How can the challenges of navigating several different health services e.g. dealing with multiple appointments and information requests, be reduced or made easier? |
| 8 | What are the major barriers for people with mental health issues when accessing physical health services and how can these be overcome? |
| 9 | Are mental health crises affected by lack of access to physical health care? |
| 10 | How effective are Annual Reviews with GPs for people living with mental health issues in terms of promoting wellbeing and preventing physical health conditions? |
| 11 | If clinicians understand that a person is experiencing mental health issues alongside their physical health conditions, does this result in better care or faster recovery? |
| 12 | What are the most effective and accessible ways to support people living with mental and physical health issues to manage their weight? |
| 13 | Can providing healthy meals (e.g. meals on wheels) and/or supporting people to cook healthy meals (e.g. cooking coaching; access to low cost cooking equipment) help people with severe mental illness manage their weight and related physical health conditions (e.g. diabetes)? |
| 14 | would information and education about healthy eating be helpful for people and their families who are living with mental and physical issues? |
| 15 | How can access to healthy foods such as fresh fruit and vegetables be improved for people living with mental and physical health issues, who may be on low incomes and/or may find it difficult to travel to shops? |
| 16 | How can weight gain linked to medication(s) be reduced or avoided? |
| 17 | What are the best ways to support friends and family carers of people with both mental and physical health issues? |
| 18 | Would greater involvement of friends and family carers in decisions and care plans improve outcomes for people with mental and physical issues? |
| 19 | Would new specialist services for people living with severe mental health issues and long-term physical health conditions make a difference to their overall health? |
| 20 | Can technology help people with both mental and physical health issues maintain their overall health? |
| 21 | What role can Personal Assistant and third sector workers play in motivating, supporting and coaching people to adopt a healthier lifestyle? |
| 22 | Could regular support from a physiotherapist or personal trainer be of benefit to people living with mental and physical health issues in helping them to become more active? |
| 23 | Some people face several lifestyle challenges. What kind of tools would help clinicians take all of them into account when making a lifestyle assessment? |
| 24 | How can take up of health promotion services such as stop smoking be supported and screening be improved? |
| 25 | Is it better to enable people to tackle unhealthy lifestyle habits all together or tackle each one separately? |
| 26 | How can people be supported to look after their mental and physical health when they face high levels of deprivation and poor access to services? |
| 27 | What is the best way to support people in maintaining their physical health when they are facing the challenges of mental health issues, low motivation or struggling to concentrate or remember information? |
| 28 | Does early support/intervention in the community health people newly diagnosed with severe mental illness adopt healthier lifestyles and prevent the development of physical health conditions? |
| 29 | How can people living with mental health issues be best supported to prevent and manage long term physical health conditions e.g diabetes, cardiovascular disease? |
| 30 | How does advice on taking care of your mental and physical health need to be adapted for people who struggle with both? |
| 31 | What does good wellbeing look like and include for people with mental and physical health issues? |
| 32 | How can the social isolation experienced by people living with mental and physical health issues be reduced or better managed? |
| 33 | How can service reach the most vulnerable groups of people with mental and physical health issues (e.g. those who are homeless, those in disadvantaged communities) |
| 34 | How does the experience of living with mental health issues affect people’s ability to understand their physical health and also manage their long-term health conditions and treatment? |
| 35 | What are the links between mental health, inflammation, exercise, diet, and gut health (microbiome)? |
| 36 | How do older people experience living with mental health issues and physical health conditions and does it impact on their ability to maintain a healthy lifestyle? |
| 37 | How can health care professionals be supported in gaining a better understanding of the relationship between mental and physical health conditions in different communities and populations? |
| 38 | What are the most practical and effective ways to encourage people with mental health issues to be more active (e.g dedicated sessions, support to use public facilities ect.)? |
| 39 | What are the benefits of creating green spaces within mental health facilities? |
| 40 | How could gym facilities be made more inclusive and accessible to people with mental and physical health issues (including those with disabling conditions)? |
| 41 | What are the barriers to people with mental health issues using schemes to help with physical health issues (e.g. leisure cards, social prescribing, gym prescriptions)? |
| 42 | What are the most practical and sustainable ways to enable people with mental and physical health issues, from different communities, to spend time in the natural environment> |
| 43 | How do environmental issues such as air pollution and noise affect people with both mental and physical health issues? |
| 44 | What information or support do clinicians and service users need to make shared decisions about managing treatments for mental and physical health conditions? |
| 45 | How much is known about the potential for physical health medications to make mental health symptoms worse? |
| 46 | How can over-prescribing medications and prescribing medications that react negatively with one another be reduced or eliminated? |
| 47 | What is the best way to support people with severe mental illness when they are diagnosed with a long-term physical health conditions (e.g diabetes, hypertension)? |
| 48 | What are the best ways to provide emotional support for people living with a mental health condition and a severe physical health condition? |
| 49 | Can effective pain management improve peoples' mental health? |
| 50 | What are the most practical and sustainable ways to treat sleep problems experienced by people with both mental and physical health issues? |
| 51 | How often and in what circumstances do physical health care services assume that a person’s experience of physical health symptoms is due to their mental health issues? |
| 52 | How good is the care of physical health problems on acute psychiatric wards (e.g. medication regimes/interactions, wound care)? |
| 53 | How can acute psychiatric wards meet the accessibility needs for people with disabling physical health conditions? |
| 54 | How can self-management support for long term physical health conditions be made more accessible to people living with mental health issues? |
